# Extra Facial Landmark Localization via Global Shape Reconstruction

**DOI:** 10.1155/2017/8710492

**Published:** 2017-04-23

**Authors:** Shuqiu Tan, Dongyi Chen, Chenggang Guo, Zhiqi Huang

**Affiliations:** School of Automation Engineering, University of Electronic Science and Technology of China, No. 2006, Xiyuan Ave, West Hi-Tech Zone, Chengdu 611731, China

## Abstract

Localizing facial landmarks is a popular topic in the field of face analysis. However, problems arose in practical applications such as handling pose variations and partial occlusions while maintaining moderate training model size and computational efficiency still challenges current solutions. In this paper, we present a global shape reconstruction method for locating extra facial landmarks comparing to facial landmarks used in the training phase. In the proposed method, the reduced configuration of facial landmarks is first decomposed into corresponding sparse coefficients. Then explicit face shape correlations are exploited to regress between sparse coefficients of different facial landmark configurations. Finally extra facial landmarks are reconstructed by combining the pretrained shape dictionary and the approximation of sparse coefficients. By applying the proposed method, both the training time and the model size of a class of methods which stack local evidences as an appearance descriptor can be scaled down with only a minor compromise in detection accuracy. Extensive experiments prove that the proposed method is feasible and is able to reconstruct extra facial landmarks even under very asymmetrical face poses.

## 1. Introduction

Facial landmark localization is the first and a crucial step for many face analysis tasks such as face recognition [1], cartoon facial animation [2, 3], and facial expression understanding [4, 5]. Most facial landmarks are located along the dominant contours around facial features like eyebrows, nose, and mouth. Therefore facial landmarks on a face image jointly describe a face shape which lies in the shape space [6].

For the last ten years remarkable progress has been made in the field of facial landmark localization [7, 8]. Among a large number of proposed methods, the most popular solution is to treat the facial landmark localization problem as a holistic shape regression process and to learn a general regression model from labeled training images [9, 10]. Following this shape regression idea, various methods try to model a regression function that directly maps the appearance of images to landmark coordinates without the need of computing a parametric model. All facial landmarks in a shape are iterated collectively and the relationship between facial landmarks is flexibly embedded into the iteration process. On the other hand, to generate enough description of face images, multiscale local feature descriptors are typically adopted in most shape regression methods. For example, cascaded pose regression (CPR) [7] was first proposed to estimate general object poses with pose-indexed features and then extended for the problem of face alignment in explicit shape regression (ESR) [11] method. ESR combines two-level boosting regression, shape-indexed features, and correlation-based feature selection. As another example, supervised descent method (SDM) [12] and its extensions also have shown an impressive performance in the field of facial landmark localization. These kinds of methods stack shape-indexed high dimension feature descriptors and train regression functions from a supervised gradient descent view.

However, facial landmark localization still meets great challenges in practical applications, such as handling pose variations and partial occlusion while maintaining moderate training model size and computational efficiency. In SDM and its improved methods, the dimension of regression matrix in each stage keeps the same with the stacked feature descriptors, where the magnification of dimension is relevant to the number of feature points. This substantially limits the use of feature descriptors with high dimension. In this type of methods, solving such high dimensional regression matrices occupies a large memory and the computation of both training and testing is not very efficient.

**Figure 1:**
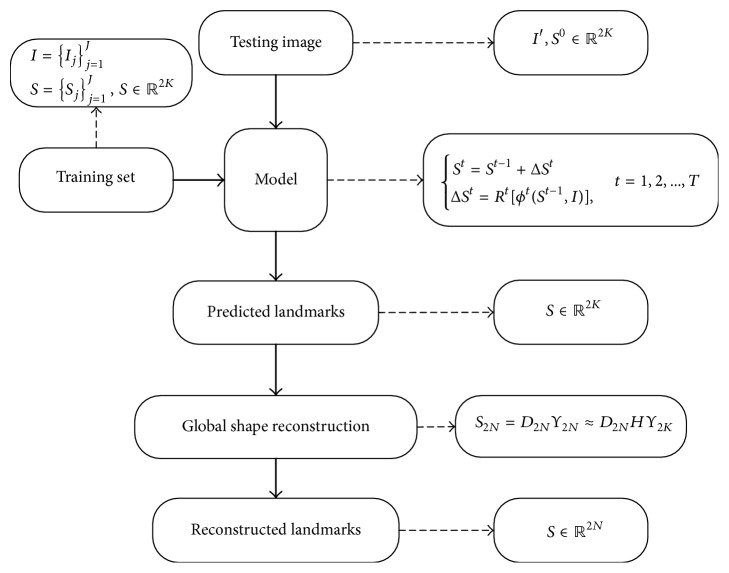
The flowchart of the proposed GSR method.

In this paper, we consider the possibility of only training a subset of available facial landmarks while in predicting stage the “missing” landmarks are restored by the inner shape correlation as if they were trained before. In this way, both the training time and the model size can be scaled down by only compromising a minor detection accuracy. Following this idea we propose a global shape reconstruction (GSR) method for predicting extra facial landmarks with limited models; see Figure 1. The concept of the proposed method is similar to the linear interpolation between facial landmarks. But there still exists a major difference since the shape prior information is adopted, which allows the proposed method to restore facial landmarks under asymmetric poses. Before the training stage, a set of shapes are collected and aligned based on the face bounding box. Then a sparse shape dictionary is trained to obtain more compact exemplars with inner shape correlation. Regressors such as SDM are trained on datasets with reduced facial landmarks. Global shape constraints are introduced on the final output of cascade regression. From another perspective, previous works that are designed or trained for fixed number of facial landmarks can interpolate more landmarks by applying proposed shape constraints.

The main contributions of this paper are the following: (1) we exploit sparse shape constraints for nonlinear restoring extra facial landmarks comparing to landmarks used for training. Specifically, we propose two strategies, named constrained global shape reconstruction with Procrustes analysis (C-GSR-PA) and simple global shape reconstruction with Procrustes analysis (S-GSR-PA), respectively, to utilize the facial landmarks correlation in landmark reconstruction procedure. (2) We study the performance between model compression and alignment error in extensive experiments. As mentioned above, using the proposed method existing works can train models on less landmarks while predicting equivalent results, but this may introduce corresponding alignment error during reconstruction. The experimental comparison results and conclusions are given in the end.

## 2. Related Work

Facial landmark localization methods can be roughly divided into two categories [7]: generative methods versus discriminative methods. Generative methods build generative models for both the face shape and appearance. They formulate face alignment as an optimization problem and generate an appearance model instance that is fit for the test face image. Discriminative methods typically independently learn regressors for each facial point and introduce a global shape model for regularization.

The active shape model [13] and active appearance model (AAM) [14] are the most well-known family of generative methods with much follow-on works. They are statistical models that attempt to describe facial appearance variations in terms of an underlying parameter space. Recently some extensions have been proposed. Matthews and Baker proposed a project-out inverse compositional algorithm (POIC) [15] to decouple shape from appearance and showed an extreme fast convergence speed. Different from POIC that projects out the appearance variations, the simultaneous inverse compositional (SIC) [16] algorithm has shown to be more robust than POIC for generic AAM fitting. Besides SIC, the alternating inverse compositional (AIC) [17] algorithm solves two separate minimization problems, one for the shape and one for the appearance.

In contrast to generative methods, discriminative methods such as deep network based methods and cascade regression based methods have attracted much attentions in recent years. In the deep network domain, Sun et al. [18] first handle the alignment task by a three-level carefully designed convolutional network. Then deep convolutional neural network (CNN) is introduced by Zhang et al. [19]. Zhou et al. [20] design a four-level convolution network cascade in a coarse-to-fine manner. Lai et al. [21] propose an end-to-end CNN architecture to learn discriminative shape-indexed features. Cascade regression is introduced into face alignment by Cao et al. [11] in their work. They design a two-level framework by again investigating boosting regression as the stage regressor. Robust cascade pose regression (RCPR) [22] is proposed to include occlusion information during learning and to pool robust features by indexing pixel features between facial landmarks. SDM [12] proposed by Xiong and De La Torre presents a cascaded linear regression formulation derived from a statistical approximation of Newton optimization. Then SDM is extended into a global optimization method called global SDM [23] to avoid a possibly local minimum. Random subspace SDM [24] is another extension and is proposed to handle the underdetermined issue and the overfitting problem in SDM.

As discussed in ESR [11] and dual sparse constrained cascade regression (DSC-CR) [25], local evidence is only sufficiently strong for a few prominent facial landmarks, such as the corner of face parts. But most of the others are not sufficiently conspicuous and cannot be reliably characterized by their image appearance. Therefore shape constraints should be explicitly introduced. Unlike the DSC-CR using a heuristic manner, we directly incorporate the sparse shape constraints into a linear regression function. In the current literatures, almost all the methods aim to design a robust feature mapping architecture or a specific form of stage regressor. In this paper, we explore a way to train models on a reduced landmark configuration and then reconstruct desired extra facial landmarks in the testing stage.

## 3. Global Shape Reconstruction Method

In this section, we present the proposed method for extra facial landmark localization in detail. First we give a brief review of the popular SDM framework. Then the training process on reduced facial landmarks is described. Finally, extra facial landmarks are predicted via proposed sparse shape constraints. An approximate sparse shape reconstruction method is also presented for reconstructing just a small number of extra facial landmarks.

### 3.1. SDM

The shape *S* of a face image is here defined by a vector of facial landmarks coordinates in *ℝ*^2*N*^ shape space; that is, *S* = (**x**_1_, **x**_2_,…, **x**_**N**_) ∈ *ℝ*^2*N*^, where *N* is the number of landmarks and **x**_**n**_ is the 2*D* coordinates of the *n*th landmark. Given an image *I* and an initial shape *S*^0^, SDM progressively refines the face shape by estimating a shape increment Δ*S*, which is added up to the shape estimate of previous stage. This can be formulated as

(1) St=St−1+ΔStΔSt=RtϕtSt−1,I,t=1,2,…,T,

where *R*^*t*^[·] is the stage regressor. In the case of SDM, *R*^*t*^[·] is generalized into a linear regressor *R*^*t*^. *ϕ*^*t*^(·) is a nonlinear feature mapping function that stacks shape-indexed feature descriptors into a single vector and *T* is the total number of regression stages. Since the feature mapping function *ϕ*^*t*^(·) is related to the previous shape estimate, the output of the function is also referred to shape-indexed features. In the training stage, the stage regressors (*R*^1^,…, *R*^*T*^) are learned sequentially.

### 3.2. Training on Reduced Facial Landmarks

Previous works mostly focus on designing a robust feature mapping function or a specific form of stage regressors [7]. Practically, we found the SDM method has a great demanding memory due to the operation of stacking feature descriptors. This substantially limits the use of high dimensional discriminative feature descriptors. In this type of methods, solving such high dimensional regression matrices occupies a large memory and the computation of both training and testing is not very efficient. Therefore we propose a method to train models on reduced facial landmarks and then localize extra facial landmarks in the testing stage. Another benefit that comes with the proposed method is that it is able to generate more constrained outputs with a given model. Note that a specific form of stage regressor for model training is not the emphasis, and here we adopt the SDM method to serve as a training framework.

Given a set of *J* labeled face images *I* = {*I*_*j*_}_*j*=1_^*J*^, along with their ground truth locations of facial landmarks *S* = {*S*_*j*_}_*j*=1_^*J*^, where *S*_*j*_ is in the *ℝ*^2*N*^shape space, *K* facial landmarks are selected out of the original *N* landmarks, which means *S*_*j*_ = (**x**_1′_, **x**_2′_,…, **x**_**K**_) and makes *S*_*j*_ in the *ℝ*^2*K*^ shape space. Let *ϕ*^*t*^(·) extract shape-indexed features on the newly formed shape *S*_*j*_. The SDM method seeks an optimal *R*^*t*^ at each stage, which minimizes the following fitting cost:

(2) argmin⁡Rt ∑jSj−Sjt−1−RtϕtSjt−1,Ij2,

where *S*_*j*_^*t*−1^ is the shape estimate of *j*th image at *t* − 1 stage. Then the shape estimate of the next stage is updated by *S*_*j*_^*t*^ = *S*_*j*_^*t*−1^ + *R*^*t*^*ϕ*^*t*^(*S*_*j*_^*t*−1^, *I*_*j*_). Equation (2) is very similar to the minimization function in RSSDM, but the regression target is different. In RSSDM, the subspaces are formed by random selecting features in the whole feature space while regressing the whole shape. However in this paper the optimization function is regressed in the shape subspace.

### 3.3. Extra Facial Landmark Reconstruction

Motivated by sparse shape constraint proposed in DSC-CR [25], we adopt the sparse signal representation to reconstruct extra facial landmarks; that is, *S*_2*K*_ ∈ *ℝ*^2*K*^ → *S*_2*N*_ ∈ *ℝ*^2*N*^  (*N* > *K*). Although the restoring problem is ill-posed, the sparse shape representation demonstrates robustness in regularizing this problem. To be more precise, let *D*_2*N*_ ∈ *ℝ*^2*N*×*M*^ denote an overcomplete shape dictionary containing *M* exemplar shapes (*M* > 2*N*). That is, any face shape *S*_2*N*_ ∈ *ℝ*^2*N*^ can be reconstructed as *S*_2*N*_ = *D*_2*N*_*γ*, where *γ* ∈ *ℝ*^*M*^ is a coefficient vector with a few nonzero entries. Since the dictionary *D*_2*N*_ is overcomplete, the above equation is underdetermined and has a unique solution if *γ* is sparsest. As for the shape of reduced facial landmarks *S*_2*K*_, a similar sparse decompose equation can be formulated as *S*_2*K*_ = *D*_2*K*_*γ*. The coefficient vector of sparse decomposition is reused in extra landmarks restoration. To summarize, for every input shape *S*_2*K*_ a corresponding sparse coefficient vector is solved by using the dictionary *D*_2*K*_. The shape dictionary *D*_2*N*_ will be combined to the coefficient vector to generate the desired output *S*_2*N*_. In the following we propose two strategies to train the dual dictionaries *D*_2*K*_ and *D*_2*N*_.

#### 3.3.1. Constrained Global Shape Reconstruction (C-GSR)

We call this a constrained strategy since the sparse coefficient vector is reused in a regressive manner. A number of face shapes *S*_2*N*_ is collected for training the dictionary *D*_2*N*_. The dictionary *D*_2*N*_ can be obtained by minimizing the following equation:

(3) argmin⁡D2N,Υ2N S2N−D2NΥ2N22,s.t. Υ2N0≤α.

In practice, we use the *k*-singular value decomposition (*K*-SVD) [26] and orthogonal matching pursuit (OMP) [27] to solve the *l*^0^-norm problem in (3). As long as the desired coefficients are sufficiently sparse, signals can also be recovered by instead minimizing the *l*^1^-norm form. Obviously, the dictionary *D*_2*K*_ can be acquired the same as (3). However in practice, we find that a simpler way to get a dictionary *D*_2*K*_ is to pool and normalize corresponding dimensions of *D*_2*N*_. More specifically, let *D*_2*N*_ = [*d*_1_^*T*^, *d*_2_^*T*^,…,*d*_2*N*_^*T*^]^*T*^ denote a set of row vectors *d*_*i*_^*T*^, *i* ∈ 1,2,…, 2*N*; we can construct the dictionary *D*_2*K*_ = [*d*_1′_^*T*^, *d*_2′_^*T*^,…,*d*_2*K*_^*T*^]^*T*^ by pooling a subset rows of *D*_2*N*_ using indexes of the *K* landmarks. This is quite straightforward because each atom in the dictionary actually represents a face shape, and the pooling operation just extracts a subshape for the same face. Then the atoms in dictionary *D*_2*K*_ should be normalized to unit length. The coefficients *Υ*_2*K*_ are calculated from

**Figure 2:**
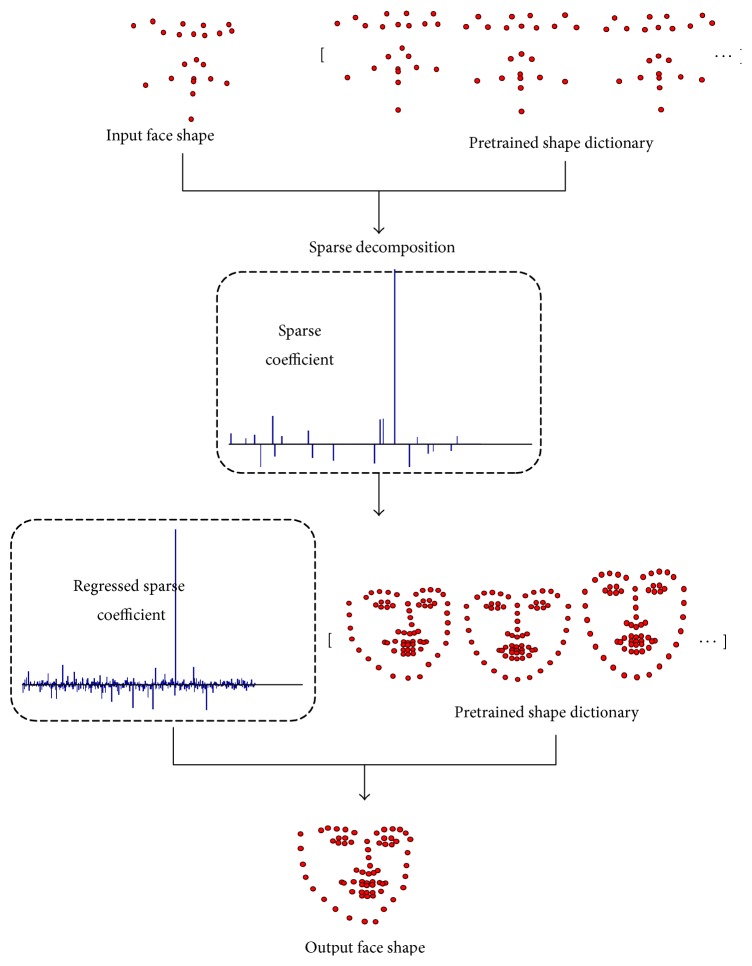
The proposed C-GSR method.

(4) argmin⁡Υ2K S2K−D2KΥ2K22,s.t. Υ2K0≤β,

where *α* and *β* are sparsity degrees, respectively. Again we utilize the OMP to solve (4). The goal is to make *Υ*_2*K*_ and *Υ*_2*N*_ as close as possible; thus a linear regression error is minimized by the following equation:

(5) argmin⁡H Υ2N−HΥ2K22,

where *H* denotes the linear regression matrix to be optimized. We apply the support vector regression (SVR) realized in LIBLINEAR toolbox [28] to solve (5). In the testing stage, extra facial landmarks can be reconstructed from *S*_2*N*_ = *D*_2*N*_*Υ*_2*N*_ ≈ *D*_2*N*_*HΥ*_2*K*_, as illustrated in Figure 2. To reduce the scale and translation drift in the process of C-GSR, we finally apply a Procrustes analysis between the newly reconstructed landmarks and the original selected landmarks. This is also denoted as C-GSR-PA.

#### 3.3.2. Simple Global Shape Reconstruction (S-GSR)

**Figure 3:**
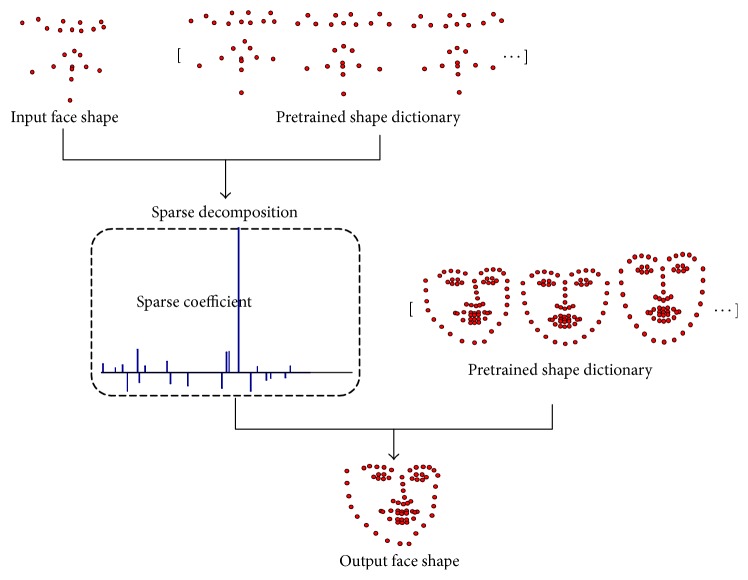
The proposed S-GSR method.

To further simplify the C-GSR method, an approximation is made as follows: after calculating all the sparse coefficient vectors in C-GSR, the sparse coefficients *Υ*_2*K*_ are considered as the rough approximation of the coefficients *Υ*_2*N*_. Then extra facial landmarks can be calculated from *S*_2*N*_ = *D*_2*N*_*Υ*_2*N*_ ≈ *D*_2*N*_*Υ*_2*K*_, when *K* → *N*, as illustrated in Figure 3. Comparing to the C-GSR strategy, S-GSR does not need to train a regression matrix in advance and requires a less computation resource. Combining with a facial occlusion detection method, S-GSR may correct a small number of occluded points based on the points of high prediction confidence.

## 4. Experiments

### 4.1. Datasets

Experiments are conducted on two datasets, namely, LFPW-68 and HELEN-68.

*LFPW-68*. The labeled face parts in the wild (LFPW) dataset [29] include 1,400 face images (1,100 images collected as the training set and the other 300 images collected as the testing set). 29 points are marked for each image. This dataset is one of the most popular datasets to benchmark facial feature detection methods in uncontrolled conditions during recent years. But in this paper, we use another version of 68 points annotations to evaluate the following experiments. Images and annotations are downloaded from the website provided by Intelligent Behavior Understanding Group [30]. And this dataset has 800 training set images and 224 testing set images.

*HELEN-68*. The HELEN dataset [31] contains 2,300 high resolutions in the wild face images and 194 points are marked for each image. All collected face images have arbitrary expressions, poses, and illumination. In this paper, we again use a version of 68 landmark annotations [30] to test the following experiments.

### 4.2. Implementation Details

As an overall setting, every training image is cropped by detecting a face bounding box and then resized to 400*∗*400 pixels and flipped together with the landmark coordinates to enlarge the training data size. To achieve a better generalization ability, 10 initial shapes are randomly sampled for each training and testing image. The cost in the SVR is set to 0.005. For the SDM method, 5 stages are used in the cascade regression. Histogram of gradient [32] is adopted as the feature descriptor with a dimension of 124. For the RCPR method, we use the default parameters reported in the original paper: 100 iterations, 15 random fern regressors, 5 restarts, and 400 features. Experiments are all running on a Laptop with an Intel Core i5-5200U CPU, a 12 GB installed memory, and a Matlab R2014a coding environment.

### 4.3. Parameters Evaluation

**Figure 4:**
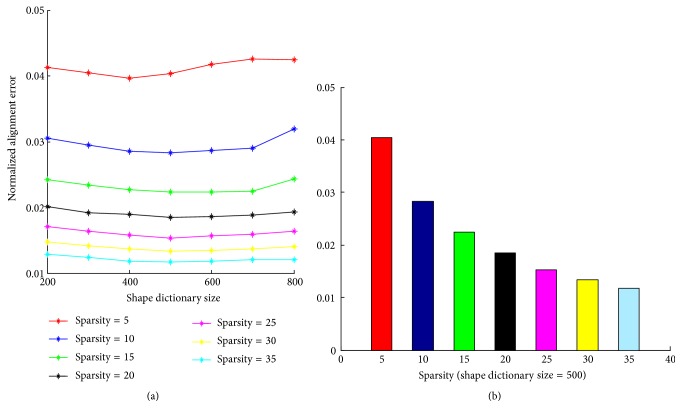
Parameters evaluation on LFPW-68 testing set.

In this section, we evaluated the reconstruction error of (3) with respect to two most important parameters, the shape dictionary size and shape sparsity, respectively, on the LFPW-68 points testing set. For the sake of simplicity, training samples can be directly normalized into atoms in the dictionary. But here *K*-SVD is utilized to train a compact shape dictionary. A grid search for two parameters is performed. The size *M* of shape dictionary is varied from 200 to 800 while keeping the shape sparsity constant. Then the shape sparsity *α* is varied from 5 to 35 while fixing the shape dictionary size. The comparing results are plotted in Figure 4. It shows that the normalized alignment error of reconstructed shapes first goes down with the growth of shape dictionary size. Then the error curves have an upward trend when shape dictionary size is larger than 500. This shows that, under a given sparsity, the size of the shape dictionary trained by *K*-SVD has an optimal value and is not the larger the better.  Figure 4(b) gives the normalized alignment error of each shape sparsity when shape dictionary size is set to 500. It shows that the alignment error monotone decreases with the shape sparsity increasing because of the greedy nature of OMP algorithm. The larger the sparsity, the smaller the reconstruction error. But the reconstruction error begins to decrease slowly after the sparsity degree exceeds 20. Considering both performance and solving speed, the turning point at sparsity 20 is chosen for both the shape sparsity degrees *α* and *β*.

### 4.4. Reconstruction Strategies Evaluation

In this section, we study the reconstruction performance under different extra facial landmarks reconstruction strategies. Two strategies of C-GSR and S-GSR with the Procrustes analysis are denoted with a “-PA” postfix. Experiments are all carried out on LFPW-68 points testing dataset; that is, *N* = 68. Here experiments are conducted on ground truth shapes since we only intend to find out the best model simplification strategy and not to test generalization ability. It will be tested in latter sections.

**Figure 5:**
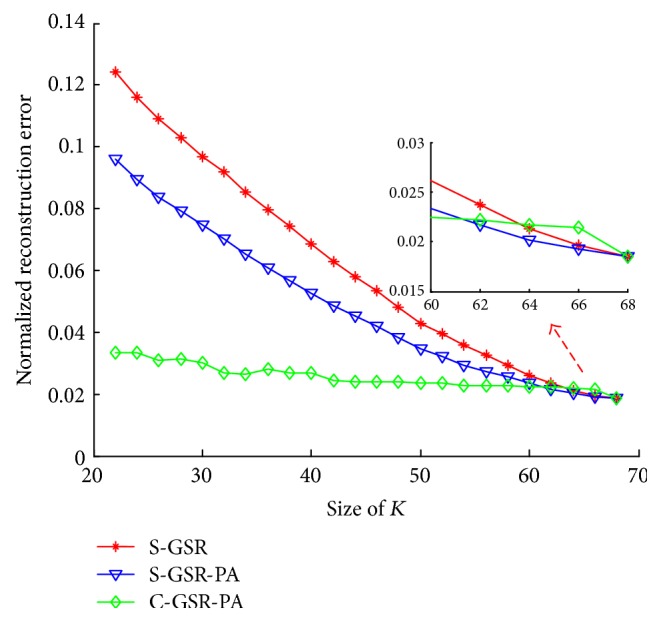
Reconstruction strategies evaluation on LFPW-68 points testing dataset.

In Figure 5, we plot the normalized reconstruction error with respect to the number of reduced landmarks, that is, the size of *K*. The size of *K* is varied from 22 to 68. We reconstruct extra facial landmarks *S*_2*K*_ ∈ *ℝ*^2*K*^ → *S*_2*N*_ ∈ *ℝ*^2*N*^  (*N* ≥ *K*) by using proposed strategies. It shows in Figure 5 that the reconstruction process has an inherent alignment error 0.0185 when *K* = *N* = 68, which means no extra landmarks are actually reconstructed. In this case, the error is introduced in the process of coefficient solving and shape synthesis. With the size of *K* growing, all the reconstruction error curves have a decrease trend. The error curve of S-GSR is obviously steeper than the error curve of C-GSR, indicating that the size of *K* has a great influence on the S-GSR method. Comparing S-GSR with S-GSR-PA, it can be found that the Procrustes analysis makes a difference when *K* is small. But it gradually plays a less role in rectifying reconstruction shifts when *K* grows larger. From the enlarged view in Figure 5 it shows that when *K* is smaller than 62 points, the C-GSR-PA achieves a far stable performance than S-GSR-PA. This is due to the rough approximation from sparse coefficients *Υ*_2*K*_ to *Υ*_2*N*_ in S-GSR-PA. But the S-GSR-PA shows a comparable performance when *K* exceeds 62 points.

**Figure 6:**
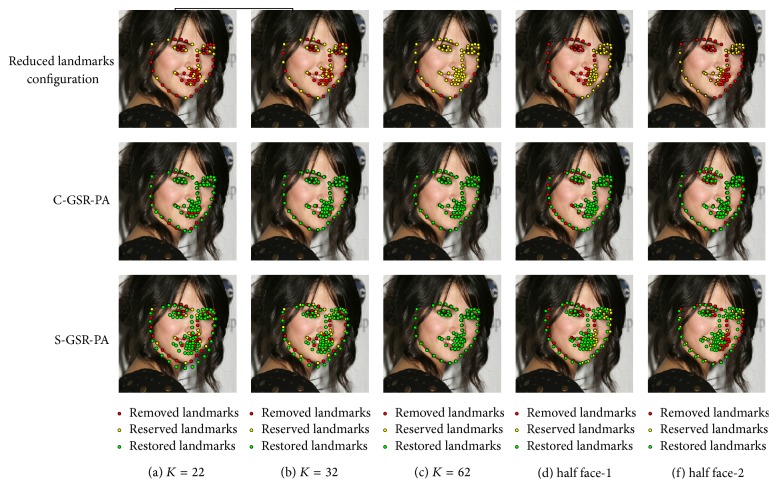
Visualization of different landmark configurations on LFPW-68 points testing dataset.

In order to get an intuitive understanding of the proposed method, we give a visualization of five different landmark configurations in Figure 6. The first row in Figure 6 illustrates the configuration pattern of reduced landmarks, and the rest two rows give the reconstruction results of C-GSR-PA and S-GSR-PA, respectively. In columns (a), (b), and (c) it is shown that more facial landmarks contribute to more accurate reconstruction performance. The S-GSR-PA has a more significant reconstruction error than the C-GSR-PA when *K* is relatively small. But if *K* is large enough, a few extra facial landmarks can be restored accurately by S-GSR-PA. To clarify the robustness of proposed method, we also illustrate the reconstruction results when half of the face landmarks are removed. This task is challenging and practical because large pose variations often exist in the wild images. It shows in columns (d) and (f) that the proposed strategies can effectively explore inner shape constraints and reconstruct the other half face landmarks even under a very asymmetry case, which is impossible for a typical linear interpolation algorithm.

### 4.5. Evaluation of C-GSR-PA on Two Facial Datasets

In this section, we evaluate the performance of the proposed method in four aspects, namely, the normalized alignment error, training time cost, testing time cost, and training model size, on two facial datasets. We first train models as described in Section 3.2. Then extra landmarks are reconstructed by proposed GSR method. SDM is chosen to serve as a cascade regression framework. In order to give an error comparison we use datasets where the number of labeled landmarks is more than 50. Therefore the alignment error can be calculated between reconstructed landmarks and labeled landmarks.

**Figure 7:**
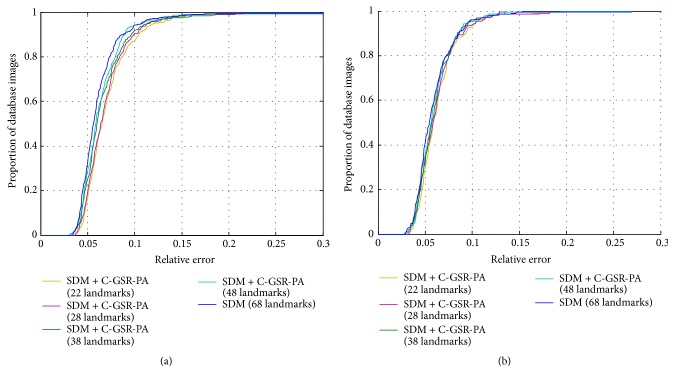
Cumulative error distribution (CED) curves of five reconstruction configurations on (a) HELEN-68 and (b) LFPW-68 datasets.

**Figure 8:**
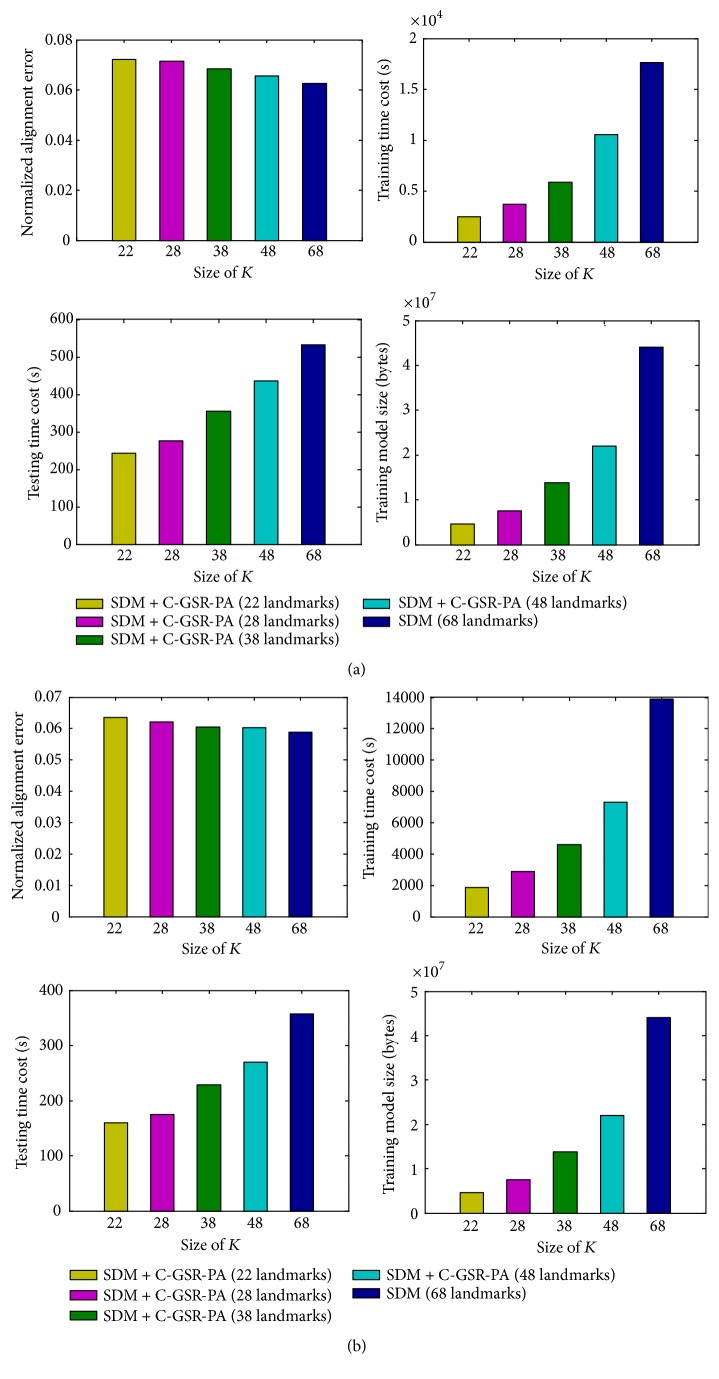
Normalized alignment error, training time cost, testing time cost, and training model size of five reconstruction configurations on two facial datasets: (a) HELEN-68 and (b) LFPW-68 datasets.

In Figure 7, we plot the cumulative error distribution (CED) curves of different reconstruction configurations. It shows in Figure 7 that using a reduced landmark configuration has a slightly less prediction accuracy than using the original SDM method. But the performance is about the same when five configurations are tested on LFPW-68 dataset. However, the performance loss in reducing landmarks at the training stage is still acceptable when considering the training time compression and model simplification. The comparing results are illustrated in Figure 8.

From Figure 8 it shows that using more landmarks for model training contributes to lower testing errors. But the difference is not significant comparing to the other three performance indicators. As is shown in Figure 8, the proposed method which trains on 22 facial landmarks and then reconstructed into 68 landmarks only has a 0.48% (LFPW-68) and 0.96% (HELEN-68) accuracy loss, comparing to the original SDM method which trains complete 68 landmarks. However, taking the experimental results of LFPW-68 dataset as an example, the method proposed in this paper has made significant progress in the following aspects compared with the original SDM method: 6.4 times faster in model training, 1.2 times faster in dataset testing, and 8.6 times smaller in model size.

**Figure 9:**
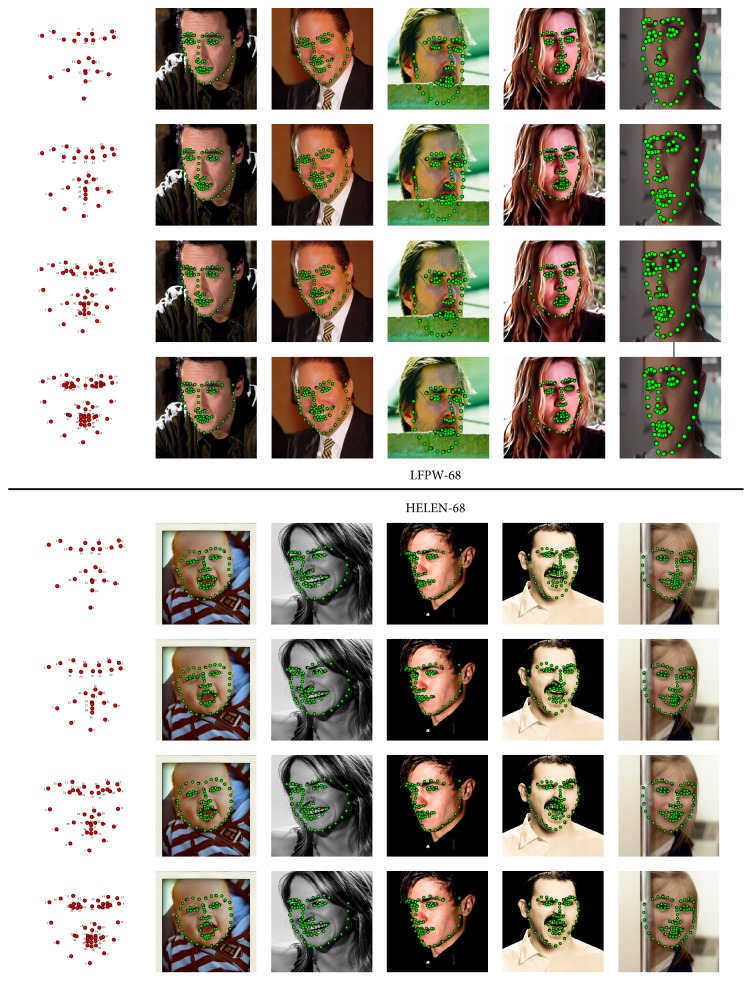
Detection results on LFPW-68 and HELEN-68 datasets. The first column gives the reduced landmark configuration for model training. For the rest of the columns, red dots on the images are the landmarks predicted by SDM and green dots on the images are the landmarks reconstructed by C-GSR-PR method.

For a better understanding, we also present the detection results with predicted landmarks labeled as green dots in Figure 9. It can be found in Figure 9 that the number of training landmarks has a positive effect on frontal images especially when the face deflection is severe. The proposed method fits well with the base points of the regression output and also gives a reconstructed shape that conforms to the contour of the face. In addition, it also shows in the figure that the landmarks located at the turning points of contours can promote the reconstruction precision. In general, by using only a 22-landmark configuration we manage to restore a 68-landmark configuration with a comparable alignment accuracy.

### 4.6. Evaluation of S-GSR-PA on Reconstructing a Small Number of Facial Landmarks

**Figure 10:**
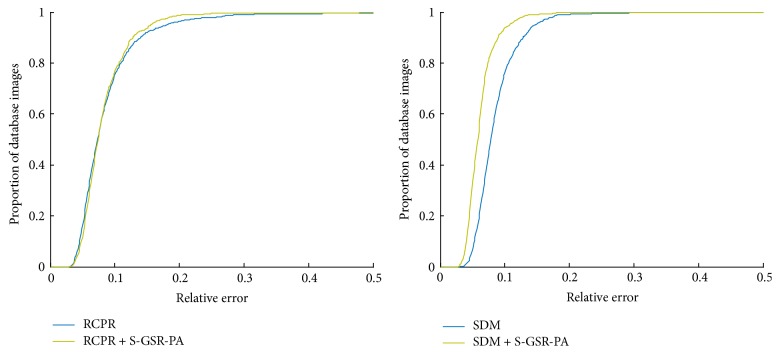
CED curves of four methods on artificial occlusion testing dataset.

As discussed in Sections 3.3.2 and 4.4, S-GSR-PA has a comparable performance on reconstructing a small number of facial landmarks. Therefore we design the following experiments and test on the LFPW-68 testing dataset. A 64 × 64 pixels' occlusion region restricted in the shape bounding box is randomly generated for each testing image. This size is selected for that only a few facial landmarks need to be occluded by the generated region. Then both the SDM and RCPR methods are tested on this artificial occlusion testing dataset for five times. Finally we run the above two methods to locate a complete facial landmark and then apply the S-GSR-PA method for refining the locations of occluded facial landmarks. Under this setting the algorithm of face occlusion detection is not discussed in this paper and the occluded regions are presumed to be known. Experimental results are plotted as CED curves in Figure 10. It shows in Figure 10 that the operation of facial landmark reconstruction helps improve the prediction accuracy of the SDM method. And it also shows a limited improvement in adjusting the RCPR method. This might be that the RCPR method can explicitly detect occlusion and use robust shape-indexed features. In general, Figure 10 clearly demonstrates that the S-GSR-PA method has a positive effect on rectifying occluded facial landmarks.

## 5. Conclusion

In this paper, we present a global shape reconstruction method for locating extra facial landmarks comparing to facial landmarks used in the training phase. By applying the proposed method, both the training time and the model size of a class of methods can be scaled down with only a minor compromise in detection accuracy. Specifically, we propose a constrained strategy (C-GSR-PA) which exploits the sparse coefficients constraints in face shape correlations. Extensive experiments show that it is able to reconstruct up to three times facial landmarks even under very asymmetrical face poses. We also propose a simplified strategy (S-GSR-PA) which shows comparable performance when reconstructing a few facial landmarks. It does not need to train a regression matrix in advance and require a less computation resource. It has potential in refining a small number of unreliable predictions.
